# Contagious accuracy norm violation in political journalism: A cross-national investigation of how news media publish inaccurate political information

**DOI:** 10.1177/14648849211032081

**Published:** 2021-07-19

**Authors:** Bartosz Wilczek, Neil Thurman

**Affiliations:** Ludwig Maximilian University of Munich, Germany

**Keywords:** Accuracy, contagion, cross-national comparative research, journalism, mis- and disinformation, social norm theory

## Abstract

This study introduces social norm theory to mis- and disinformation research and investigates whether, how and under what conditions broadsheets’ accuracy norm violation in political journalism becomes contagious and shifts other news media in a media market towards increasingly violating the accuracy norm in political journalism as well. Accuracy norm violation refers to the publication of inaccurate information. More specifically, the study compares Swiss and UK media markets and analyses Swiss and UK press councils’ rulings between 2000 and 2019 that upheld complaints about accuracy norm violations in political journalism. The findings show that broadsheets increasingly violate the accuracy norm the closer election campaigns approach to election dates. They thereby drive other news media in a media market to increasingly violate the accuracy norm as well. However, this holds only for the UK media market but not for the Swiss media market. Therefore, the findings indicate that the higher expected benefits of accuracy norm violation that exist in media markets characterised by higher competition outweigh the higher expected costs of accuracy norm violation created by stronger press councils’ sanctions, and, thereby, facilitate contagious accuracy norm violation in political journalism during election campaigns.

The spread of inaccurate information has become a serious concern for political systems. The functioning of democratic societies relies on well-informed publics (Allcott and Gentzkow, 2017; Bennett and Livingston, 2018; Coleman, 2018; Tandoc et al., 2019), and the spread of inaccurate information creates the risk that political outcomes ‘will rest on misinformation’ (Kuran and Sunstein, 1999: 736).

While inaccurate information has been classified in different ways (e.g. Bennett and Livingston, 2018; Tandoc et al., 2018), the typology of Wardle and Derakhshan (2017) has been particularly widely used in previous research. In their typology, misinformation relates to inaccurate information that is produced without the intention to harm. For instance, misinformation might consist of ‘factual errors due to unintentional or innocent mistakes’ (Ha et al., 2021: 291). Disinformation, in turn, relates to inaccurate information that is produced with the intention to harm (Wardle and Derakhshan, 2017). For instance, disinformation might be produced to shape political decisions (Hendricks and Vestergaard, 2019). However, as Ha et al. (2021) rightly argue, ‘the intention of the message is difficult to be ascertained’ (p. 291). More specifically, it is difficult to prove that actors knew the information they spread was inaccurate, and, consequently, that they spread the inaccurate information intentionally. This study therefore draws on Ha et al. (2021) and uses the term inaccurate information.

Inaccurate political information published by broadsheets, that is, supraregional up-market newspapers, may be a particular concern. Broadsheets are considered particularly important for the functioning of democracies (Hamilton, 2016). They are expected to function as ‘bouncers of the public sphere and truth’s keeper[s]’ (Hendricks and Vestergaard, 2019: xi).

Accordingly, the ‘leading thought’ (Guo and Vargo, 2020: 181) in communication science has been that broadsheets are important opinion leaders in media markets and influence the editorial decision-making of other news media (Golan, 2006; Guo and Vargo, 2020; Jarren and Vogel, 2011; Mathes and Pfetsch, 1991; Mathis and Humprecht, 2018; Shoemaker and Reese, 2011; Vonbun et al., 2016). This, in turn, suggests that if broadsheets increasingly publish inaccurate political information, other news media in a media market might rethink their own behaviour and increasingly publish inaccurate political information as well.

However, so far, these interactions have not been investigated. In fact, previous research has focused on the spread of inaccurate information on social media (e.g. Allcott et al., 2019; Burger et al., 2019; Cinelli et al., 2020; Del Vicario et al., 2016; Fletcher et al., 2018; Grinberg et al., 2019; Tandoc et al., 2020; Zhao et al., 2020). The spread of inaccurate information in news media (Guo and Vargo, 2020; Humprecht, 2019; Humprecht et al., 2020; Mourão and Robertson, 2019; Silverman, 2015; Vargo et al., 2018; Wilczek, 2020) has received less attention and is, consequently, less well understood.

Therefore, this study introduces social norm theory to mis- and disinformation research and investigates whether, how and under what conditions broadsheets’ accuracy norm violation in political journalism may become contagious and drive other news media in a media market to violate the accuracy norm in political journalism as well. In this study, accuracy norm violation refers to the publication of inaccurate information that was subsequently sanctioned by a press council. More specifically, the study compares Swiss and UK media markets and analyses Swiss and UK press councils’ rulings between 2000 and 2019 that upheld complaints about accuracy norm violations in political journalism. The study compares Swiss and UK media markets because they differ in terms of press councils’ sanctions (Fielden, 2012) as well as in the degree of competition (Picard and Russi, 2012). These media markets thereby set specific costs and benefits with regard to accuracy norm violation, which, social norm theory suggests, might affect news media’s publication of inaccurate political information.

## Contagious accuracy norm violation in political journalism

**Figure 1.**
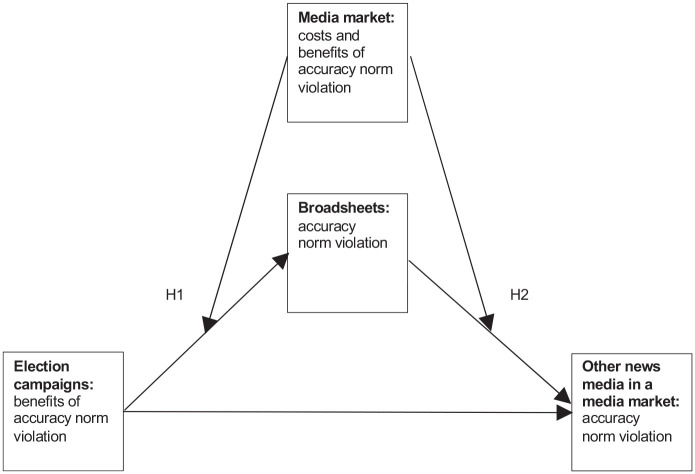
Conceptual model.

Figure 1 presents the conceptual model of this study, which draws on social norm theory and explains how and under what conditions broadsheets’ accuracy norm violation in political journalism might become contagious and drive other news media in a media market to violate the accuracy norm as well. In the following section, the theoretical building blocks are discussed, and the corresponding hypotheses are developed.

In the past several decades, social scientists have investigated how and under what conditions social norms are created, violated and enforced (Diekmann et al., 2015). Social norms are understood as statements ‘that something ought or ought not to be the case’ (Opp, 2002: 132), that is social norms are expectations about what behaviour ought to be in given situations (Axelrod, 1986). In journalism, press councils define such social norms, for instance regarding the accuracy of news. More specifically, press councils’ accuracy norms state that news media ought to publish accurate information (including political) and that they ought not to publish inaccurate information (Cohen-Almagor, 2014).

However, according to Becker (1968) as well as Diekmann et al. (2015), actors violate social norms based on cost-benefit analyses. More specifically, actors weigh the expected benefits to be gained from the norm violation against the expected costs of the norm violation created by sanctions (Gino et al., 2009). Of course, such benefits and costs will depend on the type of actors, their goals and the conditions in which they function.

Broadsheets might benefit from violating the accuracy norm in political journalism if the accuracy norm violation will serve their financial and ideological goals (Allcott and Gentzkow, 2017; Tandoc, 2019). More specifically, publishing inaccurate political information might serve broadsheets’ financial goals by attracting audiences’ attention, which, in turn, might increase their circulations and online traffic (Hendricks and Vestergaard, 2019). Furthermore, publishing inaccurate political information might serve broadsheets’ ideological goals by shaping political decisions (Hendricks and Vestergaard, 2019). For instance, broadsheets might publish inaccurate political information ‘with the intention of discrediting certain actors [. . .] [and] influencing elections’ (Tandoc, 2019: 3).

However, broadsheets will need relatively high expected benefits to be willing to violate the accuracy norm in political journalism, that is not only financial but also ideological benefits. In general, broadsheets pursue relatively high journalistic quality standards (Forschungszentrum Öffentlichkeit und Gesellschaft, 2019; Jarren and Vogel, 2011) and are expected to function as ‘truth’s keeper[s]’ (Hendricks and Vestergaard, 2019: xi). Publishing inaccurate political information may particularly serve their ideological goals during election campaigns. For instance, the closer election dates approach, the more broadsheets will try to shape election outcomes (Hameleers et al., 2019).

Accordingly, it is expected that broadsheets will increasingly violate the accuracy norm in political journalism towards elections dates. However, broadsheets’ propensity to violate the accuracy norm may depend on the media market in which they are embedded.

More specifically, broadsheets might be more likely to violate the accuracy norm in a media market that is characterised by relatively high competition (Hameleers et al., 2019). After all, the more their competitors will try to shape election outcomes, the more will broadsheets strive to achieve their own ideological goals. Moreover, ‘[c]ompetition for scoops, or to avoid being scooped, can lead to reporting without sufficient confirmation’ (Sutter, 2001: 747). Accordingly, it is expected that broadsheets in the UK will be more likely to increasingly violate the accuracy norm as election dates approach than broadsheets in Switzerland. The UK media market consists of more news media than the Swiss media market and is, therefore, characterised by higher competition (Picard and Russi, 2012).

However, according to social norm theory, norm violation can also lead to costs (Gino et al., 2009). Broadsheets face costs from violating the accuracy norm in political journalism in the form of sanctions that a press council imposes for the publication of inaccurate political information (Cohen-Almagor, 2014). The costs may involve broadsheets losing audience trust, which, in turn, may decrease their circulations and online traffic (Kalogeropoulos et al., 2019).

Depending on the media market, press councils have different sanctioning mechanisms at their disposal (Fielden, 2012; Puppis, 2009). The Swiss press council (Schweizer Presserat, 2018) is limited to publicly communicating its rulings and thereby making accuracy norm violations transparent. By contrast, the UK press councils, the Press Complaints Commission (PCC, 2014) and the Independent Press Standards Organisation (Independent Press Standards Organisation [IPSO], 2018), have also been able to force accuracy norm violators to publish corrections and adjudications. Accordingly, broadsheets in the UK will face higher costs from violating the accuracy norm in political journalism on account of stronger press councils’ sanctions, while broadsheets in Switzerland will face lower costs from violating the accuracy norm in political journalism on account of weaker press council’s sanctions.

In sum, it is assumed that higher expected benefits from violating the accuracy norm as election dates near that are created by higher competition in a media market will outweigh higher expected costs from violating the accuracy norm that are created by stronger press councils’ sanctions. Broadsheets may calculate that shaping election outcomes in a highly competitive environment will lead to relatively high benefits, while being sanctioned by a press council will result in relatively low costs. Moreover, they may assume that they will be more likely to benefit from shaping election outcomes than to experience costs due to press councils’ sanctions. After all, it is uncertain whether accuracy norm violations will lead to complaints being submitted to a press council and whether the press council will sanction the accuracy norm violation. This leads to the following hypothesis:

***H1*:** *Broadsheets will increasingly violate the accuracy norm in political journalism the closer election campaigns approach to election dates; this will hold more for the UK media market and less for the Swiss media market.*

Other news media in a media market may also benefit from violating the accuracy norm in political journalism if the accuracy norm violation serves their financial or ideological goals (Allcott and Gentzkow, 2017; Tandoc, 2019). However, compared to broadsheets, other news media (and especially tabloids and mid-market newspapers) may have lower barriers to violating the accuracy norm in political journalism. They are less constrained by expectations to function as ‘truth’s keeper[s]’ (Hendricks and Vestergaard, 2019: xi). Accordingly, they may be more willing to violate the accuracy norm to (only) achieve financial benefits (Hendricks and Vestergaard, 2019), with this willingness not confined to election periods.

However, during election campaigns, these other news media may be subject to contagious accuracy norm violation, causing them to publish more inaccurate political information as the election date approaches. Contagious norm violation occurs if knowledge about other actors’ norm violation triggers its spread (Cialdini et al., 1990; Diekmann et al., 2015). According to Gino et al. (2009), such contagion can occur in several ways. When exposed to the norm violations of others, actors may change their estimate of the likelihood of being caught violating a norm. Moreover, observing others’ norm violations may change actors’ beliefs about the appropriateness of their own actions, that is, actors may change their understanding of a norm regarding a specific behaviour.

In fact, actors are more likely to imitate other actors if these other actors are assumed to have (more) reliable information (Lemieux, 2003). This is so in part because following such experts enables actors to reduce reputational damage should a decision turn out to be wrong, that is, they can share the blame with the experts (Scharfstein and Stein, 1990). Moreover, following such experts also enables actors to reduce the costs of information search, that is, they can follow the supposedly accurate signals of experts in lieu of conducting searches themselves (Bikhchandani et al., 1992).

Accordingly, it is expected that broadsheets’ accuracy norm violation in political journalism as election dates near will become contagious and drive other news media in a media market to increasingly violate the accuracy norm as election dates approach as well. As discussed above, broadsheets are, in general, characterised by relatively high journalistic quality standards and, therefore, function as opinion leaders in media markets (e.g. Guo and Vargo, 2020; Vonbun et al., 2016).

However, the propensity of other news media to engage in contagious accuracy norm violation as election dates near may differ depending on the media market in which those news media are embedded. After all, other news media will also consider the expected benefits of violating the accuracy norm that are created not just by the approaching election date but also by the competition that exists in the media market, and weigh these against the expected costs of violating the accuracy norm that are created by a press council’s sanctions. Accordingly, it is assumed that higher expected benefits created by higher competition will outweigh higher expected costs created by stronger sanctions in the case of other news media, as well. This leads to the following hypothesis:

***H2*:** *If broadsheets increasingly violate the accuracy norm in political journalism the closer election campaigns approach to election dates, other news media in a media market will also increasingly violate the accuracy norm in political journalism; this will hold more for the UK media market and less for the Swiss media market.*

## Methods

### Data

In a first step, rulings were selected in which the Swiss and UK press councils upheld complaints about accuracy norm violations in political journalism committed by news media located in the German-speaking part of Switzerland and in the English part of the UK between January 2000 and December 2019. More specifically, rulings were considered that related to national, regional and local political news coverage. In the UK, the Independent Press Standards Organisation (IPSO) replaced the Press Complaints Commission (PCC) in 2014 (Ramsay and Moore, 2019); therefore, in the UK, the rulings of both press councils were analysed.

Moreover, while the Swiss (Schweizer Presserat, 2018) and UK (IPSO, 2018; PCC, 2014) press councils have defined accuracy similarly, they have structured their codes of practice differently. Therefore, the specific codes and rulings of the Swiss and UK press councils were considered (Table 1). The rulings were retrieved via the Swiss and UK press councils’ websites, downloaded and saved in the research database. This resulted in overall *N* = 93 rulings (i.e. cases of accuracy norm violations in political journalism): *N* = 21 rulings in the German-speaking part of Switzerland; *N* = 72 rulings in England. An overview of the cases is presented in the supplemental material.

**Table 1.** Investigated codes and rulings of the Swiss and UK press councils.

	Switzerland	UK	
Investigated press councils	Schweizer Presserat	PCC	IPSO
Investigated codes of practice	‘Erklärung der Pflichten und Rechte der Journalistinnen und Journalisten’	‘Editors’ Code of Practice’	‘Editors’ Code of Practice’
Investigated codes	Code 1: ‘Sie halten sich an die Wahrheit ohne Rücksicht auf die sich darauf für Sie ergebenden Folgen und lassen sich vom Recht der Öffentlichkeit leiten, die Wahrheit zu erfahren.’	Code 1: ‘The Press must take care not to publish inaccurate, misleading or distorted information or images, including headlines not supported by the text.’	Code 1: ‘The Press must take care not to publish inaccurate, misleading or distorted information or images, including headlines not supported by the text.’
	Code 3: ‘[. . .] Sie unterschlagen keine wichtigen Elemente von Informationen und entstellen weder Tatsachen, Dokumente, Bilder und Töne noch von anderen geäusserte Meinungen.’		
	Code 7: ‘[. . .] Sie unterlassen anonyme und sachlich nicht gerechtfertigte Anschuldigungen.’		
Investigated rulings	Complaint is upheld (‘Beschwerde wird [. . .] gutgeheissen’; ‘[. . .] hat gegen Ziffer [. . .] verstossen’).	Complaint is upheld (‘upheld’; ‘sufficient remedial action offered’).	Complaint is upheld (‘breach – sanction: publication of adjudication’; ‘breach – sanction: publication of correction’; ‘breach – sanction: action as offered by publication’).

In a second step, the rulings were analysed regarding the date when the accuracy norm violation occurred and regarding the news outlet that committed the accuracy norm violation. For the analysis, a codebook was developed and pre-tested (Neuendorf, 2017). Moreover, press councils’ rulings were coded on a monthly basis (i.e. from January 2000 until December 2019), which resulted in *N* = 480 observations, that is *N* = 240 per country. Therefore, based on this analysis, the monthly number of accuracy norm violations in political journalism was determined for broadsheets and other news media in the Swiss and UK media markets.

In Switzerland, the rulings related to the following broadsheets: *Tages-Anzeiger* (3 rulings) and *SonntagsZeitung* (1 ruling). In the UK, the rulings related to these broadsheets: *The Daily Telegraph* (13 rulings), *The Guardian* (1 ruling), *The Independent* (1 ruling) and *The Times* (6 rulings), as well as the *Sunday Telegraph* (3 rulings) and the *Sunday Times* (3 rulings).

Moreover, in Switzerland, the rulings related to the following other news media: the tabloids *Blick* (3 rulings) and *SonntagsBlick* (3 rulings), the regional newspapers *Basler Zeitung* (1 ruling), *Kreuzlinger Nachrichten* (1 ruling) and *Der Landbote* (1 ruling) and the news outlets *Facts* (1 ruling), *OnlineReports* (1 ruling), *Schweizerzeit* (1 ruling), *Weltwoche* (3 rulings), *Die Wochenzeitung* (1 ruling) and *Zeit-Fragen* (1 ruling). In the UK, the rulings related to the following other news media: the tabloids and mid-market newspapers *The Daily Express* (12 rulings), the *Daily Mail* (4 rulings), the *Daily Mirror* (4 rulings), the *Daily Star* (1 ruling), *Metro* (1 ruling), the *News of the World* (1 ruling), and *The Sun* (8 rulings), as well as the *Mail on Sunday* (2 rulings), the regional newspapers the *Bournemouth Daily Echo* (1 ruling), the *Evening Standard* (1 ruling), the *Oxford Mail* (1 ruling), the *Richmond and Twickenham Times* (1 ruling), the *Swindon Advertiser* (1 ruling) and the *Witney Gazette* (1 ruling), and the news outlets *The JC* (5 rulings) and the *New Statesman* (1 ruling).

### Measurement

#### Independent variable

The independent variable relates to election campaigns (M = 3.92; SD = 5.748). The proximity to election dates was measured based on an 18-point scale: 1 month before election = 18; 18 months before election = 1. This was determined by the fact that in the investigated countries, pre-election opinion polls were conducted over the course of this time frame. Non-election periods were coded with = 0. The dates of the national parliamentary elections were determined via websites of the Swiss and UK parliaments.

#### Mediator

The mediator relates to the monthly number of accuracy norm violations in political journalism that were committed by broadsheets in the respective media markets. This was determined via press councils’ rulings (see above) and measured based on a metric scale (M = 0.06; SD = 0.278).

#### Moderator

The moderator relates to the media market (M = .50; SD = .501). In the UK (= 1), news media have faced higher costs of accuracy norm violation on account of stronger sanctions imposed by the press council (IPSO, 2018; PCC, 2014) and expected higher benefits of accuracy norm violation on account of higher competition (Picard and Russi, 2012). By contrast, in Switzerland (= 0), news media have faced lower costs of accuracy norm violation on account of weaker sanctions imposed by the press council (Schweizer Presserat 2018) and expected lower benefits of accuracy norm violation on account of lower competition (Picard and Russi, 2012).

#### Dependent variable

The dependent variable relates to the monthly number of accuracy norm violations in political journalism that were committed by other news media in the respective media markets. This was determined via press councils’ rulings (see above) and measured based on a metric scale (M = .13; SD = .552).

#### Control

News media’s propensity to violate the accuracy norm in political journalism might increase over time as media markets get more disrupted and newsroom resources are increasingly cut (Wilczek, 2019). Moreover, in the UK, the Press Complaints Commission (PCC) was replaced in 2014 by the Independent Press Standards Organisation (IPSO) (Ramsay and Moore, 2019). Accordingly, in the UK, the number of accuracy norm violations in political journalism that have been processed and sanctioned might have increased as well. Therefore, the year (2000 = 0; 2019 = 19) in which the accuracy norm violations in political journalism were committed was controlled (M = 9.50; SD = 5.772).

The descriptive statistics are summarised in Table 2, while the bivariate correlations are indicated in Table 3.

**Table 2.** Descriptive statistics.

	Total		CH		UK	
	*M*	SD	*M*	SD	*M*	SD
Election campaigns	3.92	5.748	3.56	5.602	4.28	5.882
Media market: UK (ref. = CH)	0.50	0.501	0.00	0.000	1.00	0.000
Year	9.50	5.772	9.50	5.778	9.50	5.778
Accuracy norm violation: BR	0.06	0.278	0.02	0.128	0.11	0.366
Accuracy norm violation: ON	0.13	0.552	0.07	0.288	0.19	0.722
	*N* = 480		*N* = 240		*N* = 240	

M: mean; SD: standard deviation; UK: English part of the UK; CH: German-speaking part of Switzerland; BR: broadsheets; ON: other news media in a media market.

**Table 3.** Bivariate correlations.

	1	2	3	4
Election campaigns	–			
Media market: UK (ref. = CH)	0.062	–		
Year	0.148**	0.000	–	
Accuracy norm violation: BR	0.105*	0.173**	0.122**	–
Accuracy norm violation: ON	0.058	0.106*	0.171**	0.204**

UK: English part of the UK; CH: German-speaking part of Switzerland; BR: broadsheets; ON: other news media in a media market.

* *p* < 0.05. **p < 0.01. *N* = 480.

### Data analysis

In order to test the hypotheses, moderation analyses were performed with the PROCESS macro for SPSS (Hayes, 2018). The statistical significance of the moderated mediation was also examined with the PROCESS macro for SPSS (Hayes, 2018). For that purpose, a dual-stage moderated mediation model was chosen. Accordingly, the moderator was included in both stages of the mediation, that is, in the a- and b-paths. Moreover, to examine the moderating effect of the media market on the relationship between election campaigns and other news media’s accuracy norm violation in political journalism, the moderator was also included in the c-path. A moderated mediation is significant when the 95% confidence interval does not cross zero (Hayes, 2018).

## Findings

As Table 4 shows, the media market significantly and positively moderates the relationship between election campaigns and broadsheets’ accuracy norm violation in political journalism (*B* = 0.011, *t*(475) = 2.578, *p* = 0.010). More specifically, election campaigns are significantly and positively related to broadsheets’ accuracy norm violation in the UK media market (*B* = 0.010; *p* = 0.001) but not in the Swiss media market (*B* = −0.001; *p* = 0.834). Therefore, H1 is supported.

**Table 4.** Moderation analyses.

	*B*	SE	*t*	*p* Value
Mediator variable model				
Election campaigns	−0.001	0.003	−0.210	0.834
Media market: UK (ref. = CH)	0.049	0.030	1.644	0.101
Election campaigns × media market	0.011	0.004	2.578	0.010
Year	0.005	0.002	2.387	0.017
Constant	−0.030	0.028	−1.057	0.291
Model	*R*^2^ = 0.069, *F*(4, 475) = 8.798, *p* < 0.001. *N* = 480.			
Dependent variable model				
Election campaigns	−0.001	0.006	−0.147	0.883
Accuracy norm violation: BR	−0.143	0.269	−0.529	0.597
Media market: UK (ref. = CH)	0.042	0.059	0.715	0.475
Election campaigns × media market	0.006	0.009	0.657	0.512
BR × media market	0.536	0.285	1.878	0.061
Year	0.014	0.004	3.241	0.001
Constant	−0.056	0.056	−0.995	0.320
Model	R^2^ = 0.076, *F*(6, 473) = 6.489, *p* < 0.001. *N* = 480.			

UK = English part of the UK; CH = German-speaking part of Switzerland; BR = broadsheets.

Moreover, as Table 4 further shows, the media market also significantly and positively moderates the relationship between broadsheets’ accuracy norm violation in political journalism and other news media’s accuracy norm violation in political journalism (*B* = 0.536, *t*(473) = 1.878, *p* = 0.061). More specifically, broadsheets’ accuracy norm violation is significantly and positively related to other news media’s accuracy norm violation in the UK media market (*B* = 0.393; *p* < 0.001) but not in the Swiss media market (*B* = −0.143; *p* = 0.597). Therefore, H2 is also supported.

Furthermore, as the index of the moderated mediation shows (Table 5), the moderated mediation is significant (*Index* = 0.004, 95% CI = <0.001–0.012). This confirms that broadsheets, as election dates neared, increasingly violated the accuracy norm in political journalism, which drove other news media in the media market to increasingly violate the accuracy norm as election dates neared as well. However, as the conditional indirect effects show (Table 5), this holds only for the UK media market (*Index* = 0.004, 95% CI = <0.001–0.012) but not for the Swiss media market (*Index* = <0.001, 95% CI = −0.001 to 0.001), which is in line with H1 and H2.

**Table 5.** Moderated mediation analysis.

	Index	SE	CI LL	CI UL
Index of moderated mediation				
	0.004	0.003	<0.001	0.012
Conditional indirect effects				
Media market: CH	0.000	0.000	−0.001	0.001
Media market: UK	0.004	0.003	<0.001	0.012
	*B*	SE	*t*	*p* Value
Conditional direct effects				
Media market: CH	−0.001	0.006	−0.147	0.883
Media market: UK	0.005	0.006	0.788	0.431

*N* = 480.

CH: German-speaking part of Switzerland; UK: English part of the UK.

Finally, as Table 5 further shows, election campaigns have no significant direct effect on other news media’s accuracy norm violation in political journalism, either in the UK media market (*B* = 0.005, *p* = 0.431) or in the Swiss media market (*B* = −0.001, *p* = 0.883). Therefore, broadsheets’ accuracy norm violation in political journalism fully mediates the relationship between election campaigns and other news media’s accuracy norm violation in political journalism. However, as indicated above, this holds only for the UK media market.

## Discussion

This study introduced social norm theory to mis- and disinformation research and investigated how broadsheets’ accuracy norm violation in political journalism becomes contagious and drives other news media in a media market to violate the accuracy norm as well. In this study, accuracy norm violation refers to the publication of inaccurate information that is sanctioned by a press council. The study compared Swiss and UK media markets because they differ in terms of press councils’ sanctions (Fielden, 2012) as well as in the degree of competition (Picard and Russi, 2012), which, social norm theory suggests, might affect news media’s publication of inaccurate political information.

The findings show that broadsheets increasingly violate the accuracy norm during election campaigns, that is, they publish more inaccurate political information the closer campaigns approach to election dates. Broadsheets’ accuracy norm violation, in turn, becomes contagious and drives other news media in a media market to increasingly violate the accuracy norm during election campaigns as well. However, this holds only for the UK media market, which is characterised by stronger press council’s sanctions (IPSO, 2018; PCC, 2014) and higher competition (Picard and Russi, 2012), but not for the Swiss media market, which is characterised by weaker press council’s sanctions (Schweizer Presserat, 2018) and lower competition (Picard and Russi, 2012).

More specifically, the findings highlight the importance of broadsheets regarding the emergence of inaccurate information in political journalism. First, the findings suggest that broadsheets are particularly strategic in their violations of the accuracy norm, that is, they publish more inaccurate political information during election campaigns, which amplify the ideological benefits of inaccurate political information. This is in line with research that shows the closer election dates approach, the more broadsheets will compete in order to shape election outcomes (Hameleers et al., 2019).

Second, by increasingly violating the accuracy norm during election campaigns, broadsheets incentivise other news media in a media market to increasingly violate the accuracy norm during election campaigns as well. This does not mean that other news media do not pursue ideological goals. However, the findings suggest that other news media are more likely to publish inaccurate political information (which might also have ideological benefits) during election campaigns under the condition that broadsheets are willing to publish more inaccurate political information. This may be so in part because, when exposed to the accuracy norm violations of broadsheets, other news media may change their estimate of the likelihood of being caught violating the accuracy norm (Gino et al., 2009). Moreover, observing broadsheets’ accuracy norm violation may change other news media’s beliefs about the appropriateness of their own actions (Gino et al., 2009).

However, this holds only for the UK media market but not for the Swiss media market. This may be explained by the fact that, while news media in the UK have faced higher costs of accuracy norm violation on account of stronger press council’s sanctions, they have also expected higher benefits of accuracy norm violation on account of higher competition. By contrast, in Switzerland, news media have faced lower costs of accuracy norm violation on account of weaker press council’s sanctions but also expected lower benefits of accuracy norm violation on account of lower competition.

Accordingly, the findings indicate that the higher expected benefits from publishing inaccurate political information that exist in conditions of higher competition in a media market outweigh the higher expected costs of publishing inaccurate political information that exist when there are stronger press council’s sanctions. This, in turn, suggests that higher competition in a media market facilitates contagious accuracy norm violation during election campaigns. It also indicates that press councils’ sanctions might not be sufficiently effective (Cohen-Almagor, 2014; Ramsay and Moore, 2019) to contain accuracy norm violation under conditions of higher competition.

## Conclusions

Previous research has focused on how inaccurate information spreads on social media. The spread of inaccurate information in news media, however, has had less attention and, accordingly, is less well understood. Therefore, this study contributes to the understanding of how and under what conditions inaccurate information emerges in political journalism. This is crucial in order to be better able to prevent the proliferation of inaccurate information.

More specifically, the findings of this study show that broadsheets increasingly publish inaccurate political information the closer election campaigns approach to election dates. This, in turn, incentivises other news media in a media market to increasingly publish inaccurate political information during election campaigns as well. The findings therefore indicate a two-step process (Katz, 1957) of accuracy norm violation during election campaigns, that is, broadsheets as opinion leaders (Guo and Vargo, 2020; Vonbun et al., 2016) shift other news media towards increasingly violating the accuracy norm as well. This is concerning, as broadsheets are expected to function as ‘bouncers of the public sphere and truth’s keeper[s]’ (Hendricks and Vestergaard, 2019: xi) and, accordingly, are considered to be particularly important for the functioning of democracies (Hamilton, 2016).

However, this two-step process of accuracy norm violation during election campaigns occurs only in in the UK media market but not in the Swiss media market. Therefore, the findings also indicate that higher competition in a media market facilitates the publication of inaccurate political information. Consequently, the findings suggest that press councils’ sanctions are not sufficiently effective (Cohen-Almagor, 2014; Ramsay and Moore, 2019) to contain accuracy norm violation under conditions of higher competition.

While it is in broadsheets’ (long-term) self-interest to ensure the accuracy of news, media accountability (Fengler et al., 2011) and media governance (Puppis, 2007) may need to play an increasingly important role in the future. Press councils have been reorganising their processes and revising their codes of practice in order to become more efficient and effective in the digital age. However, ‘media accountability and media governance [. . .] must be seen as a process of different but interrelated practices’ (Eberwein and Porlezza, 2016: 334). Accordingly, a greater diversity of actors who are involved in media accountability and media governance activities – for instance different types of fact-checkers (Amazeen, 2020; Andersen and Søe, 2020; Fletcher et al., 2020; Graves, 2018; Singer, 2020) – might facilitate the containment of inaccurate information in political journalism (Southwell and Thorson, 2015).

Several limitations of this study need to be addressed. First, the findings reveal a relationship between broadsheets’ accuracy norm violation and other news media’s accuracy norm violation during election campaigns. However, causality has still to be established. While the findings indicate that election campaigns have no direct effects on other news media’s accuracy norm violation, the influence of further factors is possible. Therefore, a promising path for future research would be to investigate this relationship under controlled conditions.

Second, the findings suggest that broadsheets function as opinion leaders in media markets and influence other news media in terms of accuracy norm violation during election campaigns. However, the findings do not reveal the actual decision-making of journalists within news organisations. For instance, based on these findings it is not possible to ascertain whether and when journalists knew that the political information they published was inaccurate (Ha et al., 2021). It could be that they were sometimes unaware that the political information was inaccurate or that they were uncertain whether it was accurate. However, the findings do suggest that news media reported the inaccurate political information without sufficient verification – due to ideological benefits (i.e. broadsheets) and contagion (i.e. other news media) under conditions of higher competition in the media market.

Therefore, a further promising path for future research would be to apply qualitative methods (e.g. in-depth interviews and observations) and to investigate whether and why journalists knowingly violate the accuracy norm. Based on such findings, ‘the intention of the message[s]’ (Ha et al., 2021: 291) could be better identified, which, in turn, would facilitate determining if the accuracy norm violation draws on mis- or disinformation (Wardle and Derakhshan, 2017). However, such qualitative approaches would have limitations as well, for instance due to the social desirability that might shape subjects’ answers and behaviour during the data collection.

Third, the study investigated two media markets, which differ in terms of press council’s sanctions and competition (Doyle, 2013), that is, Switzerland and the UK. To further examine how contagious accuracy norm violation in political journalism varies depending on sanctions (i.e. stronger vs weaker press council’s sanctions) and competition (i.e. higher vs lower competition), future research might investigate a broader sample of countries.

Fourth, the study selected the investigated cases of accuracy norm violation based on Swiss and UK press councils’ rulings that upheld complaints about violations. This allowed an investigation of clearly defined populations of accuracy norm violations. Moreover, it allowed the analysis of cases that represent particularly severe instances of accuracy norm violation. However, the investigated populations do not incorporate all the inaccurate political information that was published in the analysed media markets during the examined time frame. Accordingly, future research might sample the investigated cases of inaccurate political information based on a mix of sources, which could also include fact-checkers’ output.

Finally, the study revealed that broadsheets play a crucial opinion-leading role with regard to accuracy norm violation in political journalism as election dates near, that is, the findings show how and under what conditions broadsheets’ accuracy norm violation becomes contagious and drives other news media to increasingly violate the accuracy norm as well. However, larger sample sizes would facilitate investigating this relationship in more detail. More specifically, future research might investigate how broadsheets’ opinion leadership regarding accuracy norm violation differs depending on the type of news media influenced (e.g. tabloids vs regional news media; traditional news media vs pure online players).

## Supplemental Material

sj-pdf-1-jou-10.1177_14648849211032081 – Supplemental material for Contagious accuracy norm violation in political journalism: A cross-national investigation of how news media publish inaccurate political information Click here for additional data file. Supplemental material, sj-pdf-1-jou-10.1177_14648849211032081 for Contagious accuracy norm violation in political journalism: A cross-national investigation of how news media publish inaccurate political information by Bartosz Wilczek and Neil Thurman in Journalism
