# Supplementary material for: Contagious accuracy norm violation in political journalism: A cross-national investigation of how news media publish inaccurate political information
Source: Journalism (Lond). 2021 Jul 19;23(11):2271–88. doi: 10.1177/14648849211032081 (PMC9660276; doi:10.1177/14648849211032081)
Supplement: sj-pdf-1-jou-10.1177_14648849211032081 – Supplemental material for Contagious accuracy norm violation in political journalism: A cross-national investigation of how news media publish inaccurate political information [file sj-pdf-1-jou-10.1177_14648849211032081.pdf]

SUPPLEMENTAL MATERIAL

Contagious accuracy norm violation in political journalism: A cross-national investigation of how news media publish inaccurate political information

Table 1: Swiss cases

News media	Dates	Cases	Press council
Zeit-Fragen	Aug-00	The article "Die Schweiz 1937 und der heutige Pilet-Golaz-Bundesrat" breached clause 1.	Schweizer Presserat
WochenZeitung	Aug-00	The article "Zur Annahme der 18-Prozent-Initiative durch die SVP" breached clause 1.	Schweizer Presserat
SonntagsBlick	Sep-00	The article "Neonazis: Sie schiessen rum und rekrutieren neue Mitglieder" breached clause 1.	Schweizer Presserat
Landbote	Mar-01	The article "Klare Inhalte - neue Töne" breached clause 1.	Schweizer Presserat
Weltwoche	Oct-02	The article "Eine Million Franken pro Flüchtling" breached clause 3.	Schweizer Presserat
Schweizer Zeit	May-03	The article "Sprachregelung" breached clause 7.	Schweizer Presserat
SonntagsBlick	Sep-04	The article "Jean-Pierre Bonny (...) über die neue Regionalpolitik von (...) Joseph Deiss" brached clause 3.	Schweizer Presserat
SonntagsBlick	Oct-04	The article "Wende im Fall Zäch" breached clause 3.	Schweizer Presserat
Facts	Dec-04	The article "Terror-Alarm bei den Sozialdemokraten"breached clause 7.	Schweizer Presserat
Kreuzlinger Nachrichten	Aug-07	The article "Wen kümmerts - Abschiedsgeschenk" breached clause 7.	Schweizer Presserat
Tages-Anzeiger	Apr-09	The article "Schweizer Demokrat droht nicht genehmen Journalisten" breached clause 7.	Schweizer Presserat
Blick	Jun-10	The article "Diese Frau hat uns den Zebrastreifen verordnet" breached clause 1.	Schweizer Presserat
Weltwoche	Jun-10	The article "Wehrli, Bremi, Leimgruber, Beyeler, Widmer-Schlumpf, Janom Steiner, Brand" breached clause 1.	Schweizer Presserat
onlinereports.ch	Aug-10	The article "Immer noch hat die PNOS ihren Hetzartikel nicht gelöscht" breached clause 1.	Schweizer Presserat
tages-anzeiger.ch	Nov-12	The article "Kantonsrat bestätigt Immunität von Mario Fehr" breached clause 1.	Schweizer Presserat
Basler Zeitung	Feb-14	The article "Auf Staatskosten mit Partnern nach Stockholm" breached clause 1.	Schweizer Presserat
Blick	Jul-14	The article "Diese Politiker gerieten auf die schiefe Bahn" breached clause 7.	Schweizer Presserat
Blick	Dec-14	The article "Sex-Skandal von Zug" breached clause 1.	Schweizer Presserat
SonntagsZeitung	Mar-18	The article "Bananenrepublik Moutier" breached clause 1.	Schweizer Presserat
Weltwoche	Oct-18	The article "Volksverächter" breached clause 3.	Schweizer Presserat
tages-anzeiger.ch	Jun-19	The article "SVP Ständerat soll Frauen mit obszönen Gesten beleidigt haben" breached clause 1.	Schweizer Presserat

Table 2: UK cases

News media	Dates	Cases	Press councils
The Sunday Times	Apr-00	The article "Labour says the old are racist" breached clause 1.	PCC
The Independent	Jun-00	The article "Blair 'all spin and little delivery'" breached clause 1.	PCC
The Times	Feb-02	The article "Blair ally leads push against Speaker" breached clause 1.	PCC
The Daily Telegraph	Jul-04	The article "Howard gets tough on 'bedblocking' MPs" breached clause 1.	PCC
The Sunday Telegraph	Oct-04	The article "Blair prepares to use his deadliest weapon" breached clause 1.	PCC
Daily Express	Jan-07	The article "Council axes Christian prayers after 600 years" breached clause 1.	PCC
News of the World	Mar-09	The article "Tory secrecy campaigner's £60k payout" breached clause 1.	PCC
The Sunday Telegraph	May-09	The article "Cash secrets of MPs who tried to stop you seeing their expenses" (1/2) breached clause 1.	PCC
The Daily Telegraph	May-09	The article "Cash secrets of MPs who tried to stop you seeing their expenses" (2/2) breached clause 1.	PCC
The Sunday Times	May-09	The article "Commons officials helped culture secretary beat tax" breached clause 1.	PCC
The Daily Telegraph	Jun-09	The article "Tory claims £57,000 to rent flat from own company" breached clause 1.	PCC
The Guardian	Jan-10	The article "Niece of British MP admits stabbing lover while high on drugs" breached clause 1.	PCC
The Sun	Jan-10	The article "Minister's niece on murder trial" breached clause 1.	PCC
The Evening Standard	Jan-10	The article "Minister's niece slashed throat of lover then told police: I am a monster" breached clause 1.	PCC
Daily Mail	Jan-10	The article "MP's sex killer niece is jailed for 15 years" breached clause 1.	PCC
Daily Mirror	Jan-20	The article "Quentin Davies' niece sentenced to 15 years for murder" breached clause 1.	PCC
Daily Star	Jul-10	The article "Muslim-only public loos" breached clause 1.	PCC
New Statesman	Sep-10	The article "Cameron, the Tea Party and a little backbench problem" breached clause 1.	PCC
The Sunday Telegraph	Jul-11	The article "Inside story of Murdoch's special relationship with our politicians" breached clause 1.	PCC
The Daily Telegraph	Oct-11	The article "Lutfur Rahman: all his controversies in one place" breached clause 1.	PCC
Daily Express	Nov-14	The article "Ukip is now more popular than Labour" breached clause 1.	IPSO
The Daily Telegraph	Mar-15	The article "Stuttering start to Clegg's campaign" breached clause 1.	IPSO
The Daily Telegraph	Apr-15	The article "Sturgeon's secret backing for Cameron" breached clause 1.	IPSO
The Times	Apr-15	The article "Labour's £1,000 tax on families" breached clause 1.	IPSO
Bournemouth Echo	Jul-15	The article "Councils learning from conviction" breached clause 1.	IPSO
The Daily Telegraph	Aug-15	The article "Labour grandees round on 'anti-Semite' Corbyn" breached clause 1.	IPSO
The Sun	Sep-15	The article "Court Jezter" breached clause 1.	IPSO
The Daily Telegraph	Sep-15	The article "Why should I pay for Jeremy Corbyn's friend Claire to have so many children?" breached clause 1.	IPSO
The Sun	Jun-16	The article "Senior council worker suspended for sickening homophobic response (…) " breached clause 1.	IPSO
Daily Express	Jul-16	The article "98% say no to EU deal" breached clause 1.	IPSO
The Mail on Sunday	Jul-16	The article "'Baroness Brazen' under fire for handing £90,000 to Labour crony's firm (…) " breached clause 1.	IPSO
express.co.uk	Aug-16	The article "Sturgeon's flagship 'Named Person' scheme savaged by dad kept from sick baby" breached clause 1.	IPSO
The Times	Oct-16	The article "Jews blamed for Holocaust at 'shameful' House of Lords event" breached clause 1.	IPSO
The Daily Telegraph	Dec-16	The article "(…) But all this is now under threat from MPs and Lords'" breached clause 1.	IPSO
The Times	Feb-17	The article "Police to investigate Glasgow corruption" breached clause 1.	IPSO
express.co.uk	Aug-17	The article "Seven in 10 Britons now support hard Brexit according to major survey" breached clause 1.	IPSO
The Daily Telegraph	Aug-17	The article "Boost for May as remainers back a hard exit" breached clause 1.	IPSO
mirror.co.uk	Aug-17	The article "Theresa May sets date she'll quit as Prime Minister (…) " breached clause 1.	IPSO
The Sunday Times	Feb-18	The article "Labour welcomes back banned activists and Holocaust denier" breached clause 1.	IPSO
thesun.co.uk	Feb-18	The article "Labour welcomes banned radical accused of anti-Semitism a (…) " breached clause 1.	IPSO
The Jewish Chronicle	Feb-18	The article "Labour readmits member following 'antisemitic' remarks on Jews and Zionism" breached clause 1.	IPSO
Daily Mail Online	Feb-18	The article " (…) member among the farleft activists who have been let back into Labour (…) " breached clause 1.	IPSO
express.co.uk	Feb-18	The article "Holocaust denier and hard-leftists readmitted into Labour Party (…)" breached clause 1.	IPSO
Daily Mirror	Feb-18	The article "You're off your trolley" breached clause 1.	IPSO
The Sun	Feb-18	The article "Mum jibe at PM" breached clause 1.	IPSO
Witney Gazette	Feb-18	The article "Mayor set to hand over chains after months of controversy" breached clause 1.	IPSO
Oxford Mail	Feb-18	The article "Controversial Carterton mayor Lynn Little forced to hand over chain" breached clause 1.	IPSO
Richmond and Twickenham Times	May-18	The article "Mayor's last event before handing over her chain of office to successor" breached clause 1.	IPSO
The Times	Jun-18	The article "Menopause suicide" breached clause 1.	IPSO
Daily Mail Online	Jun-18	The article "Widowed ex-Mayor, 55, hanged herself at home (…)" breached clause 1.	IPSO
Daily Mirror	Jun-18	The article "Suicide over menopause" breached clause 1.	IPSO
The Times	Jul-18	The article "Terror police boost MP's security over criticism of Asian sex gangs" breached clause 1.	IPSO
Swindon Advertiser	Aug-18	The article "Suspended Ukip man posting on party's website" breached clause 1.	ISPO
express.co.uk	Oct-18	The article "Corbyn accused of wanting to 'brainwash school kids into hating UK' (…) " breached clause 1.	IPSO
express.co.uk	Oct-18	The article "What border problems? MP tweets photo of Swiss border with just ONE sign" breached clause 1.	IPSO
The Sun	Oct-18	The article "£4BN FOREIGN AID TO FIX POTHOLES IN INDIA" breached clause 1.	IPSO
The Daily Telegraph	Jan-19	The article "The British people won't be scared into backing a woeful Brexit deal (…)" breached clause 1.	IPSO
express.co.uk	Jan-19	The article "Theresa May warns of DANGEROUS results of second Brexit referendum" breached clause 1.	IPSO
The Jewish Chronicle	Feb-19	The article "Ex-Militant Tendency activist accused of bullying Louise Ellman (…)" breached clause 1.	IPSO
The Jewish Chronicle	Mar-19	The article "Top activist: 'Only hate is Jews vs Jews'" breached clause 1.	IPSO
The Jewish Chronicle	Apr-19	The article "'Vile' attacks by soap star condemned" breached clause 1.	IPSO
express.co.uk	May-19	The article "Scottish nationalists CLASH with pro-Union activists (…)" breached clause 1.	IPSO
The Sun	May-19	The article "Go with the BoJo or end up like dodo" breached clause 1.	IPSO
The Daily Telegraph	Jun-19	The article "The one move that will transform the life chances of a generation" breached clause 1.	IPSO
The Mail on Sunday	Jun-19	The article "Corbyn's 'war on homeowners'" breached clause 1.	IPSO
Daily Mail	Jul-19	The article "Labour tax raid on family homes" breached clause 1.	IPSO
express.co.uk	Jul-19	The article "Labour's inheritance tax horror plan to raid 10 million Britons' life savings" breached clause 1.	IPSO
metro.co.uk	Jul-19	The article "Labour tax plan 'could stop parents passing on homes to kids" breached clause 1.	IPSO
The Daily Telegraph	Aug-19	The article "Public backs Johnson to shut down Parliament for Brexit" breached clause 1.	IPSO
express.co.uk	Sep-19	The article "Brexit betrayal: Johnson launches ruthless bid (…)" breached clause 1.	IPSO
The Sun	Nov-19	The article "Corb's tax bombshell would cost the average Sun reader an extra... £2,400" breached clause 1.	IPSO
The Jewish Chronicle	Dec-19	The article "Call for Green candidates to be suspended" breached clause 1.	IPSO
